# GDF11 inhibits adipogenesis and improves mature adipocytes metabolic function via WNT/β‐catenin and ALK5/SMAD2/3 pathways

**DOI:** 10.1111/cpr.13310

**Published:** 2022-08-03

**Authors:** Jan Frohlich, Kristina Kovacovicova, Marco Raffaele, Tereza Virglova, Eliska Cizkova, Jan Kucera, Julie Bienertova‐Vasku, Martin Wabitsch, Marion Peyrou, Francesca Bonomini, Rita Rezzani, George N. Chaldakov, Anton B. Tonchev, Michelino Di Rosa, Nicolas Blavet, Vaclav Hejret, Manlio Vinciguerra

**Affiliations:** ^1^ International Clinical Research Center St. Anne's University Hospital Brno Czech Republic; ^2^ Psychogenics Inc Tarrytown New York USA; ^3^ Research Center for Toxic Compounds in the Environment (RECETOX) Masaryk University Brno Czech Republic; ^4^ Faculty of Medicine, Department of Pathological Physiology Masaryk University Brno Czech Republic; ^5^ Division of Pediatric Endocrinology and Diabetes, Department of Pediatrics and Adolescent Medicine University of Ulm Ulm Germany; ^6^ Departament de Bioquímica i Biomedicina Molecular and Institut de Biomedicina Universitat de Barcelona Barcelona Spain; ^7^ Centro de Investigación Biomédica en Red "Fisiopatología de la Obesidad y Nutrición" Madrid Spain; ^8^ Institut de Recerca Hospital Sant Joan de Déu Barcelona Spain; ^9^ Anatomy and Physiopathology Division, Department of Clinical and Experimental Sciences University of Brescia Brescia Italy; ^10^ Interdepartmental University Center of Research "Adaption and Regeneration of Tissues and Organs‐(ARTO)", University of Brescia Brescia Italy; ^11^ Department of Translational Stem Cell Biology Research Institute of the Medical University Varna Bulgaria; ^12^ Department of Anatomy and Cell Biology Research Institute of the Medical University Varna Bulgaria; ^13^ Department of Biomedical and Biotechnological Sciences Human Anatomy and Histology Section, School of Medicine, University of Catania Catania Italy; ^14^ CEITEC‐Central European Institute of Technology Masaryk University Brno Czech Republic; ^15^ National Center for Biomolecular Research Masaryk University Brno Czech Republic

## Abstract

**Objective:**

GDF11 is a member of the TGF‐β superfamily that was recently implicated as potential “rejuvenating” factor, which can ameliorate metabolic disorders. The main objective of the presented study was to closely characterize the role of GDF11 signaling in the glucose homeostasis and in the differentiation of white adipose tissue.

**Methods:**

We performed microscopy imaging, biochemical and transcriptomic analyses of adipose tissues of 9 weeks old *ob/ob* mice and murine and human pre‐adipocyte cell lines.

**Results:**

Our in vivo experiments employing GDF11 treatment in *ob/ob* mice showed improved glucose/insulin homeostasis, decreased weight gain and white adipocyte size. Furthermore, GDF11 treatment inhibited adipogenesis in pre‐adipocytes by ALK5‐SMAD2/3 activation in cooperation with the WNT/β‐catenin pathway, whose inhibition resulted in adipogenic differentiation. Lastly, we observed significantly elevated levels of the adipokine hormone adiponectin and increased glucose uptake by mature adipocytes upon GDF11 exposure.

**Conclusion:**

We show evidence that link GDF11 to adipogenic differentiation, glucose, and insulin homeostasis, which are pointing towards potential beneficial effects of GDF11‐based “anti‐obesity” therapy.

## Graphical abstract

GDF11 activates distinct signaling pathways in pre‐adipocytes (β‐catenin) and in mature adipocytes (WNT pathway), to regulate adipogenesis and adiponectin secretion/insulin sensitivity, respectively.

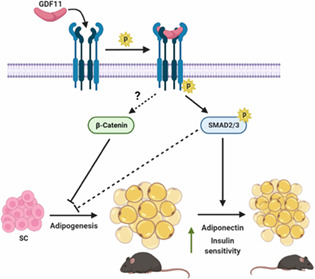

## 1 INTRODUCTION

White adipose tissue (WAT) is a connective tissue composed of adipocytes and stromal vascular cells, including immune cells. During the past few decades, there has been a dramatic change of perspective towards WAT. This tissue is no longer considered as inert mass of tissue serving as energy storage and insulation, but it is one of the most important endocrine organs of the human body that releases many biologically active compounds collectively designated adipokines (leptin, adiponectin, visfatin and many others).^1^, ^2^, ^3^, ^4^ In normal conditions or during calorie restriction, adipocytes supplement nutrients to bodily tissues through lipolysis and their sensitivity to glucose/insulin as well as production of adipokines is optimal.^5^ Upon nutritional excess and physical activity deficit, adipocytes accumulate more fat, loose sensitivity to insulin and secrete less metabotrophic adipokines (e.g., adiponectin, nerve growth factor/NGF, and brain‐derived neurotrophic factor/BDNF), which leads to severe outcomes like atherosclerosis, metabolic syndrome and/or type 2 diabetes mellitus (T2DM).^1^, ^3^, ^6^ When the fat storage capacity is exceeded, fat starts to accumulate in other sites like liver and skeletal muscle, which promotes metabolic consequences of obesity and increased mortality.^7^, ^8^ In order to compensate for the lack of fat storage, new adipocytes are formed from adipose tissue mesenchymal stem cells,^9^, ^10^ and newly formed adipocytes are more insulin sensitive, thus helping ameliorating insulin insensitivity.^11^The process of adipogenesis is a tightly regulated cellular process with many signaling pathways involved^12^, ^13^ and, among those, TGF‐β signaling has well‐established regulatory roles in the adipogenic process. There is more than 30 human TGF‐β superfamily ligands that can be classified, based on their structure and function, into two distinct subfamilies: TGF‐βs and BMPs.^14^ BMP ligands signal through SMAD1/5/8 (R‐SMADs) are of particular interest, since some members (BMP2, BMP4 and BMP7) have been shown to promote adipogenic differentiation of precursor cells into the adipose lineages with high efficiency.^15^, ^16^, ^17^, ^18^, ^19^ In contrast to BMPs, TGF‐β ligands (such as TGF‐β and myostatin) signal via intracellular SMAD2/3 complexes, which inhibit adipocyte commitment by decreasing C/EBPs and PPARγ expression in bone marrow stem cells (BMSCs) and mesenchymal stem cells (MSC).^20^, ^21^, ^22^ Ablation or inhibition of TGF‐β signaling in MSC results in a marked expansion of adipocytes in mice.^23^

Growth differentiation factor 11 (GDF11) is a member of the TGF‐β subfamily with pleiotropic roles during the mammalian embryonic development. Its expression is characterized by variable intensity in different tissues, including heart, skeletal muscle, nervous system, kidney, pancreas and intestine.^24^ During the past decade, GDF11 has been heralded as a powerful “anti‐aging” factor or “fountain of youth”, having the ability to reverse age‐related phenotypes in rodents, rejuvenating cardiac and skeletal muscle,^25^, ^26^ improving systemic glucose homeostasis,^27^ expanding the brain vasculature and improve cognitive functions.^3^, ^28^, ^29^ Nonetheless, others have disputed the age‐reversal properties of GDF11 and doubted its rejuvenating properties in aged rodents.^30^, ^31^, ^32^, ^33^, ^34^ Furthermore, the latter studies also showed that elevated levels of GDF11 have deleterious effects on aging skeletal muscle regeneration and that supra‐physiological doses of GDF11 can promote cachexia and premature death.^35^, ^36^, ^37^

Recently, it was reported that *Gdf11* gene transfer or recombinant GDF11 protein supplementation in rodents ameliorated high‐fat diet (HFD) induced obesity, hyperglycemia, insulin resistance, nonalcoholic fatty liver disease (NAFLD) and obesity.^38^, ^39^, ^40^ GDF11 triggered calorie restriction‐like phenotype and stimulated secretion of adiponectin from WAT.^41^ Also, GDF11 treatment inhibited adipogenic differentiation of mouse and human pre‐adipocytes by impinging SMAD2/3‐dependent TGF‐β pathway.^42^These recent data highlight anti‐adipogenic effects of GDF11, but the underlying mechanisms of action remain incompletely understood. The main aim of the present study was to specifically characterize the role of GDF11 of signaling in WAT metabolism and adipogenic differentiation in vitro and in vivo, using a powerful combination of imaging, transcriptomic, biochemical and molecular biology approaches.

## 2 MATERIALS AND METHODS

### 2.1 Cell culture

Stable 3T3‐L1 pre‐adipocytes were cultured and differentiated into mature adipocytes according to an established protocol, and at the 10th day of differentiation they were processed for further analyses.^43^, ^44^ For microscopy analyses, 3T3‐L1 cells were seeded on coverslips. Simpson–Golabi–Behmel syndrome (SGBS) pre‐adipocyte cells were obtained, cultured, and differentiated as previously described.^45^, ^46^, ^47^

### 2.2 Mice models

The use of *ob/ob* mice complied with the institutional and European legislation concerning vivisection, the use of genetically modified organisms, animal care and welfare (European Directive 2010/63/UE adopted by the European Parliament and the Council of the EU on September 22, 2010). The granted experimental protocol n°516/2018‐PR was approved by the University of Brescia Institutional Animal Care Committee (Brescia, Italy) and was conducted in accordance with national and European regulations. *ob/ob* mouse lines were maintained on a C57BL/J6 background within the University of Brescia animal facility (Brescia, Italy), in temperature‐controlled rooms under a 12 h light/dark cycle, in conventional cages with enriched environment and standard diet. Mice had access to food and water ad libitum. For experimental purposes mice were divided into two groups of 9 weeks old *ob/ob* mice (*n* = 12 per cohort) and were injected daily by i.p. injection at 7 p.m. (in order to ensure that circulating GDF11 concentration reached its peak during the active nocturnal phase of mice) for two consecutive weeks with either saline (controls, *ob/ob*) or GDF11 (0.1 mg/kg, *ob/ob* rGDF11) (Supplementary Figure 1).

### 2.3 Microscopy and fluorescence imaging

Cells seeded on coverslips (either 3T3‐L1 or SGBS) were differentiated according to the appropriate standard protocol^44^ and, after successful differentiation, coverslips were washed with PBS and fixed with 4% paraformaldehyde for 10 min at room temperature. After fixation and further washings with PBS, cells were stained with either Oil Red O solution in 40% isopropanol or BODIPY lipid staining dye (1 μg/ml) for 30 min. Coverslips were then mounted on microscope slides with Gelatin (1%) mounting medium containing DAPI (1 μg/ml), and images were captured using an Axio scan Z.1 (Zeiss) equipped with a Hamamatsu ORCA‐Flash 4.0 camera and ImageJ software analysis program (NIH Image, Bethesda, MD) was used to evaluate all immunofluorescence images. When grown in multi‐well plates (24 wells), cells stained with ORO or BODIPY were measured by spectrophotometer ThermoScientific Multiscan GO or by fluorescence measurement using Biotek FLX800 equipped with appropriate fluorescence filters (DAPI 360/460 ex/em; BODIPY 480/520 ex/em), respectively.

### 2.4 Histological and immunofluorescence analyses

Samples of murine epidydymal WAT (eWAT) and interscapular brown adipose tissue (BAT) were embedded and snap frozen in Tissue Freezing Media (Leica Microsystems, Wentzler, Germany) and were cut to 7 μm at −20°C with a cryostat (Leica Microsystems, Wetzlar, Germany). The slides were then processed by haematoxylin & eosin (H&E) staining for histological evaluation, as described previously.^48^, ^49^, ^50^ The histological analyses methods are fully described in the supplementary information.

### 2.5 Immunoblotting analyses

Protein extraction and immunoblotting analyses were performed as previously described.^49^, ^51^, ^52^ Antibodies used in this study: Cell signaling—rabbit anti‐Adiponectin (C45B10; 1:1000), rabbit anti‐pSMAD2^(Ser467)^ (138D4; 1:1000), rabbit anti‐SMAD2/3 (D7G7; 1:1000), rabbit anti‐β‐catenin (D10A8; 1:1000) and secondary antibody goat Anti‐rabbit IgG HRP‐linked (1:2000); Abcam—mouse anti‐β‐actin HRP conjugated antibody (AC‐15; 1:2000), mouse anti‐GAPDH monoclonal HRP conjugated antibody (1:2000)

### 2.6 RT‐PCR and RNA‐sequencing

Total RNA was extracted from WAT samples of three control and GDF11‐treated *ob/ob* mice, or four samples (from two different cell line passages) per treatment group in the case of mature 3T3‐L1 cells (CTL; GDF11 100 ng/ml; and/or SB431542 100 μM, XAV939 [100 μg/ml] and IWR1 [100 μg/ml]) with TRIzol Reagent (Invitrogen, CA) and column separation using a RNeasy Mini Kit (Qiagen, Germany), according to manufacturer's instructions. DNASEI treatment was used during RNA isolation protocol to isolate pure total RNA without DNA contamination. RNA integrity was assessed using Agilent RNA 6000 Nano Kit, Agilent 2100 Bioanalyzer (both Agilent Technologies, CA) and automated electrophoresis system—TapeStation (Agilent Technologies). The RNA‐sequencing (RNA‐Seq) method is fully described in the Supplementary information. For RT‐PCR, 1 μg of total isolated RNA was used to prepare cDNA using a High‐Capacity cDNA Reverse Transcription Kit (ThermoFisher Scientific, MA). RT‐PCR was performed using StepOnePlus Real‐Time PCR System (Applied Biosystems, Darmstadt, Germany) and SYBR Select Master Mix (ThermoFisher Scientific, MA). The human and murine primer sequences used in this study are listed in Supplementary Table 1.

### 2.7 Statistical analyses

All statistical analyses were performed using GraphPad Prism Software (version 7.00 for Windows; GraphPad Inc., CA). Statistical comparisons between groups were made using the parametric Student's *t* test, if the data had normal distribution in all tested subgroups, otherwise the nonparametric Mann–Whitney *U* test was used instead. To determine statistical significance between more than two groups, a parametric One‐Way ANOVA was used when the data had a normal distribution, or otherwise a non‐parametric Kruskal–Wallis test, as appropriate. Independent experiments were carried out at least three times with three technical replicates. The data are expressed as the means ± SD (unless indicated otherwise). Differences were considered statistically significant at *p* < 0.05 (*), *p* < 0.01 (**), and *p* < 0.001 (***) or indicated otherwise.

## 3 RESULTS

### 3.1 GDF11 ameliorates insulin insensitivity and decreases white adipocyte size in *ob/ob* mice

**FIGURE 1:**
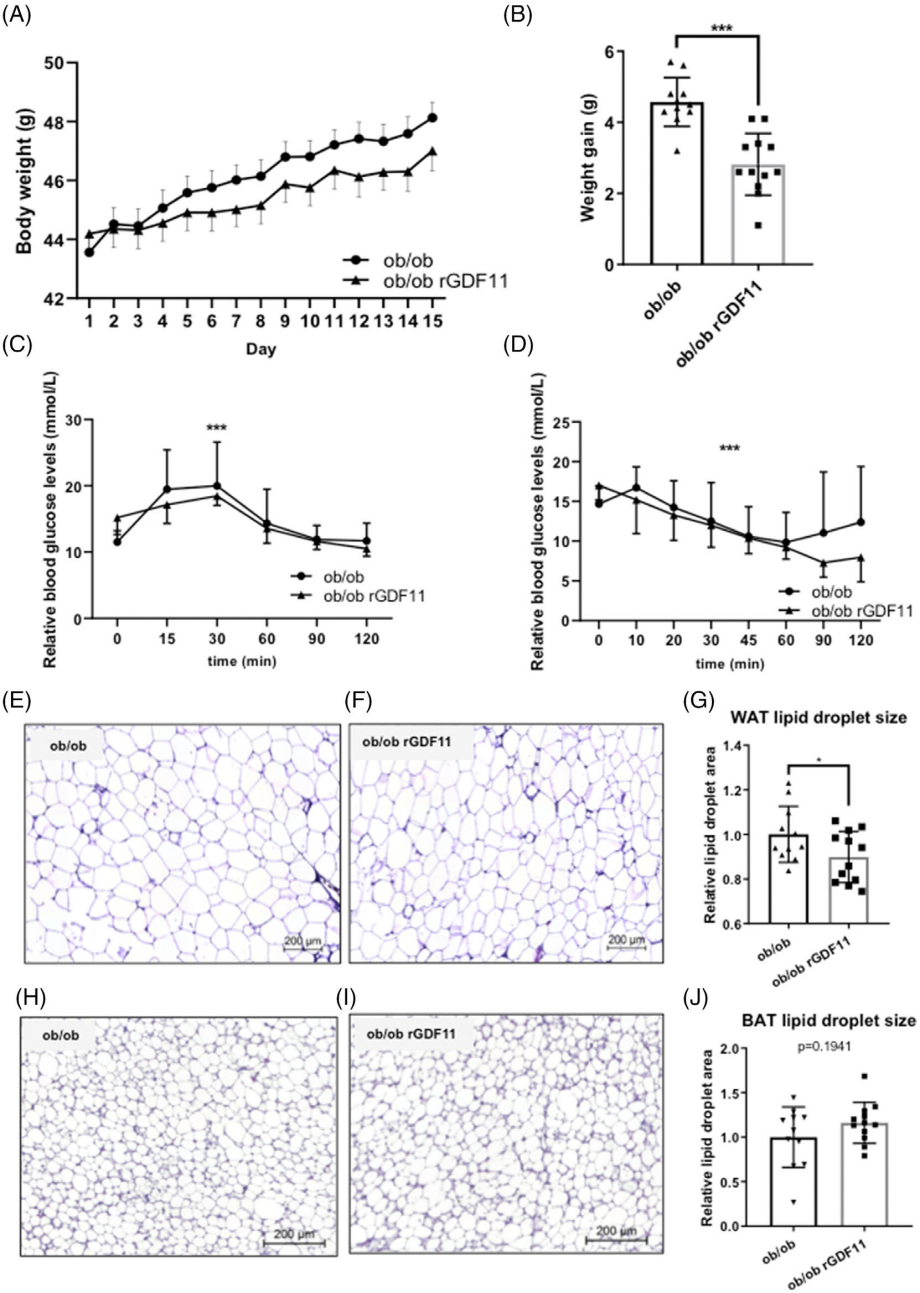
GDF11 treatment ameliorates insulin sensitivity, restores glucose homeostasis and affects lipid droplet size in *ob/ob* adipose tissues. (A) Body weight growth curves of 10‐week‐old *ob/ob* mice treated by GDF11 or saline over the period of 14 days. (B) Quantification of total weight gain of mice after 14 days experimental period (*n* = 11 per group). ***p < 0.001 (Mann–Whitney *U* test). GTT (C) and ITT (D) tests in CTL and GDF11 treated *ob/ob* mice (*n* = 11 per group). Asterisk ***p < 0.001 mean statistical significance. Representative images of HE stained epidydymal white adipose tissue (eWAT) in (E) CTL and (F) GDF11 treated *ob/ob* mice (200 μm scale). (G) Quantification of eWAT lipid droplet size (area, μm^2^) as in A and B, respectively. The whole imaged area of eWAT samples of at least 10 animals per group was evaluated. Representative images of HE stained brown adipose tissue (BAT) in (H) CTL and (I) GDF11 treated *ob/ob* mice (200 μm scale). (J) Quantification of BAT lipid droplet size (area, μm^2^) as in D and E, respectively. The whole sample image area of at least 10 animals per group was evaluated. *p < 0.05 and ***p < 0.001 (Mann–Whitney *U* test).

Since GDF11 administration can ameliorate high fat induced obesity,^39^ we sought to test its in vivo effect in micegenetic obesity model, *ob/ob* mice. Two groups of 9 weeks old *ob/ob* mice (*n* = 12 per cohort) were injected daily by i.p. injection at 7 p.m. for two consecutive weeks with either saline (controls, CTL) or rGDF11 (0.1 mg/kg) (Supplementary Figure 1). At the end of the experiment, GDF11‐treated mice were significantly leaner and gained less weight during the experiment than CTL mice (Figure 1A). Total weight gain, measured from start to end point of the experiment, was significantly higher (*p* < 0.001) in the CTL group (4.57 ± 0.68 g) than in the GDF11 group (2.82 ± 0.87 g) (Figure 1B). Also, daily chow consumption was slightly higher (*p* < 0.05) in the control group (10.95 ± 1.29 g) when compared with GDF11‐treated group (10.69 ± 1.26 g) (data not shown). Blood glucose levels after 6 h fasting were higher (*p* ≤ 0.001) in GDF11 treated group (15.50 ± 2.99 mmol/L) compared with the CTL (11.50 ± 3.95 mmol/L), but during GTT, upon injection with 1 g/kg of glucose, levels of blood glucose were increasing significantly (*p* ˂ 0.001) slower in the first 30 min and the peak was observed later in the GDF11‐treated group compared with the controls (Figure 1C). During ITT, glucose levels were decreasing more rapidly after insulin injection (1 U/kg) in the GDF11 group than in the CTL mice (Figure 1D). GDF11 administration in *ob/ob* mice leads to decreased weight gain, and to an increased systemic glucose tolerance and insulin sensitivity.Since insulin sensitivity and blood glucose levels are tightly bound to the hypertrophy of adipose tissue, we explored how GDF11 treatment affected WAT and brown adipose tissue (BAT) size in *ob/ob* mice. For this purpose, we histologically analyzed eWAT and intrascapular BAT, respectively. No differences in crude weight of isolated eWAT fat lobes in our test groups were observed (1.82 ± 0.13 g in CTL vs. 1.76 ± 0.16 g in GDF11, *p* = 0.354). However, employing H&E staining coupled to morphological assessment we observed significant difference in the average size (μm^2^) of lipid vacuoles between the CTL (5176 ± 643.51 μm^2^) and the GDF11 group (4595 ± 586.13 μm^2^) (*p* = 0.026) (Figure 1E–G). No changes were observed in the size of BAT lipid vacuoles (CTL 241.9 ± 82.19 μm^2^ and GDF11 280.9 ± 55.68 μm^2^; Figure 1H–J).

### 3.2 GDF11 triggers an anti‐adipogenic gene expression program in white adipose tissue in obese mice

**FIGURE 2:**
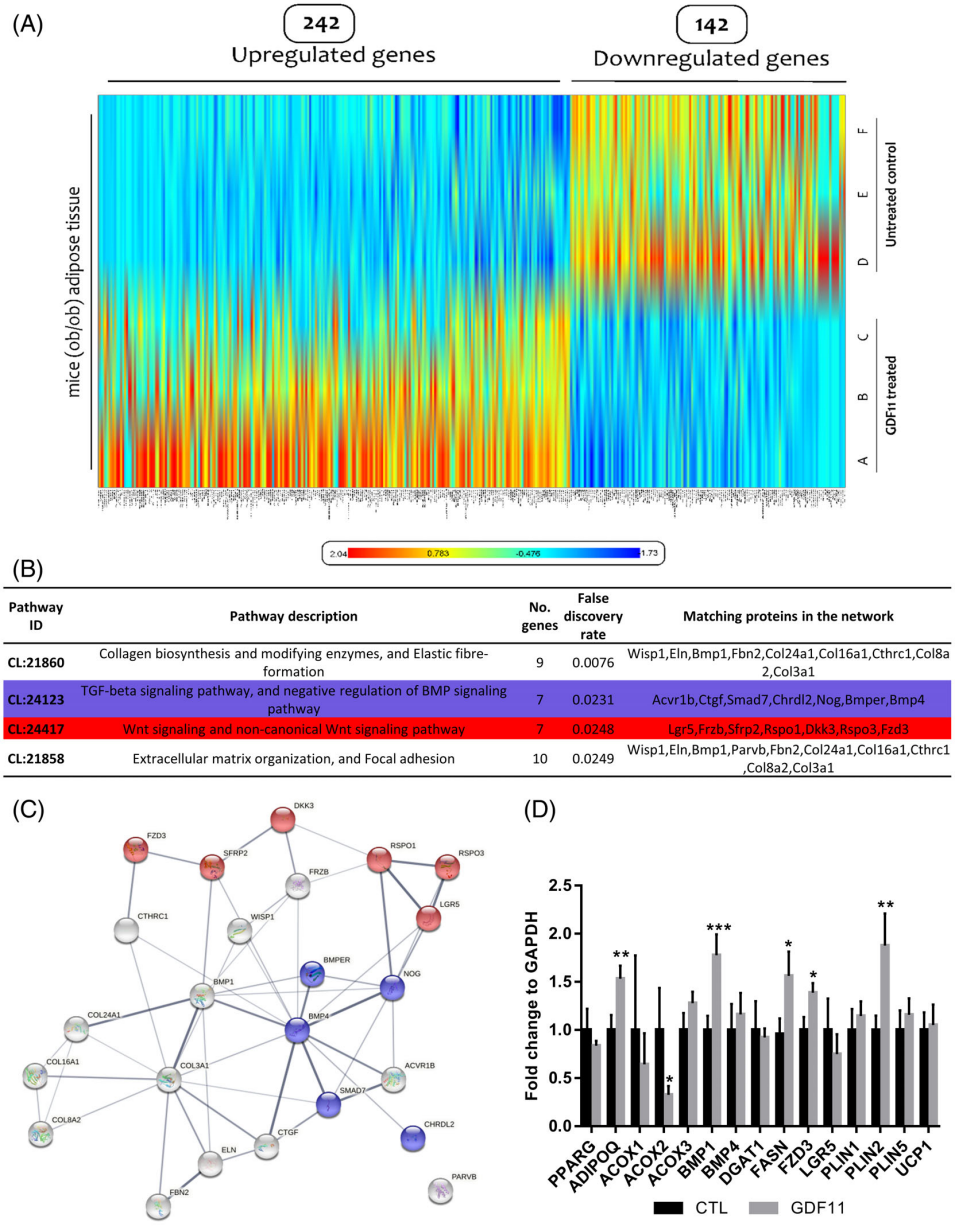
GDF11 treatment significantly affects mRNA expression levels in adipose tissue of obese mice and cooperates with WNT/β‐catenin pathway. (A) Heat map showing differences in mRNA expression levels in WAT between CTL and GDF11‐treated *ob/ob* mice (*n* = 3 per group). (B) String pathway analysis of genes involved in adipogenesis and lipogenesis in sequenced eWAT mRNA as in A. (C) Graphical representation of gene interactions by string analysis for WNT/β‐catenin and TGF‐β signaling pathways B. Connections between genes are indicating annotated, high confidence (>0.75) molecular interactions between genes. (D) mRNA expression levels of selected genes involved in TGF/WNT signaling (as in A‐B), adipocyte differentiation and fat metabolism in *ob/ob* WAT (at least *n* = 7 per treatment group). *p < 0.05; **p < 0.01; ***p < 0.001 (Mann–Whitney *U* test, compared with CTL).

To uncover signaling pathways and related gene expression patterns that might reflect changes in eWAT upon GDF11 treatment in *ob/ob* mice, we analyzed transcriptome of eWAT samples by sequencing (RNA‐Seq). GDF11 treatment had significant and vast effect on the overall gene expression in *ob/ob* eWAT (Figure 2A). In total, we were able to identify 384 differentially (*p* ˃ 0.05) expressed genes, of which 242 were over‐expressed, and 142 were downregulated (Supplementary File 1). To identify genes and signaling pathways that can clarify observed phenotype, we used STRING unbiased analysis of annotated molecular interactions.^53^ Among the top signaling pathways influenced by GDF11 treatment we identified TGF‐β signaling specific genes. Moreover, our analysis uncovered that WNT signaling specific genes were also affected by the GDF11 treatment (Figure 2B). Further analysis uncovered significant signaling overlap/crosstalk between TGF‐β and WNT pathways that was manifested by close interactions of deregulated genes belonging into those pathways (Figure 2C). To validate observed expression changes of genes involved in TGF‐β/WNT pathways and those in adipogenesis and lipid metabolism, RT‐PCR was used (Figure 2D). We observed significantly elevated levels of adiponectin, BMP1, FZD3, FASN, and PLIN2, whereas levels of ACOX2 were decreased significantly.

### 3.3 GDF11 inhibits adipogenic differentiation in 3T3‐L1 and in SGBS cells

To analyse the role of GDF11 supplementation in pre‐adipocytes, we firstly established cytotoxicity measurement of rGDF11 treatment on 3T3‐L1 pre‐adipocytes with various concentrations of GDF11 recombinant protein (25–100 ng/ml). Cell viability/proliferation was monitored using a DAPI assay. We observed that treatment with 25–100 ng/ml of rGDF11 did not have significant cytotoxic effects on 3T3‐L1 pre‐adipocytes (*data not shown*).

**FIGURE 3:**
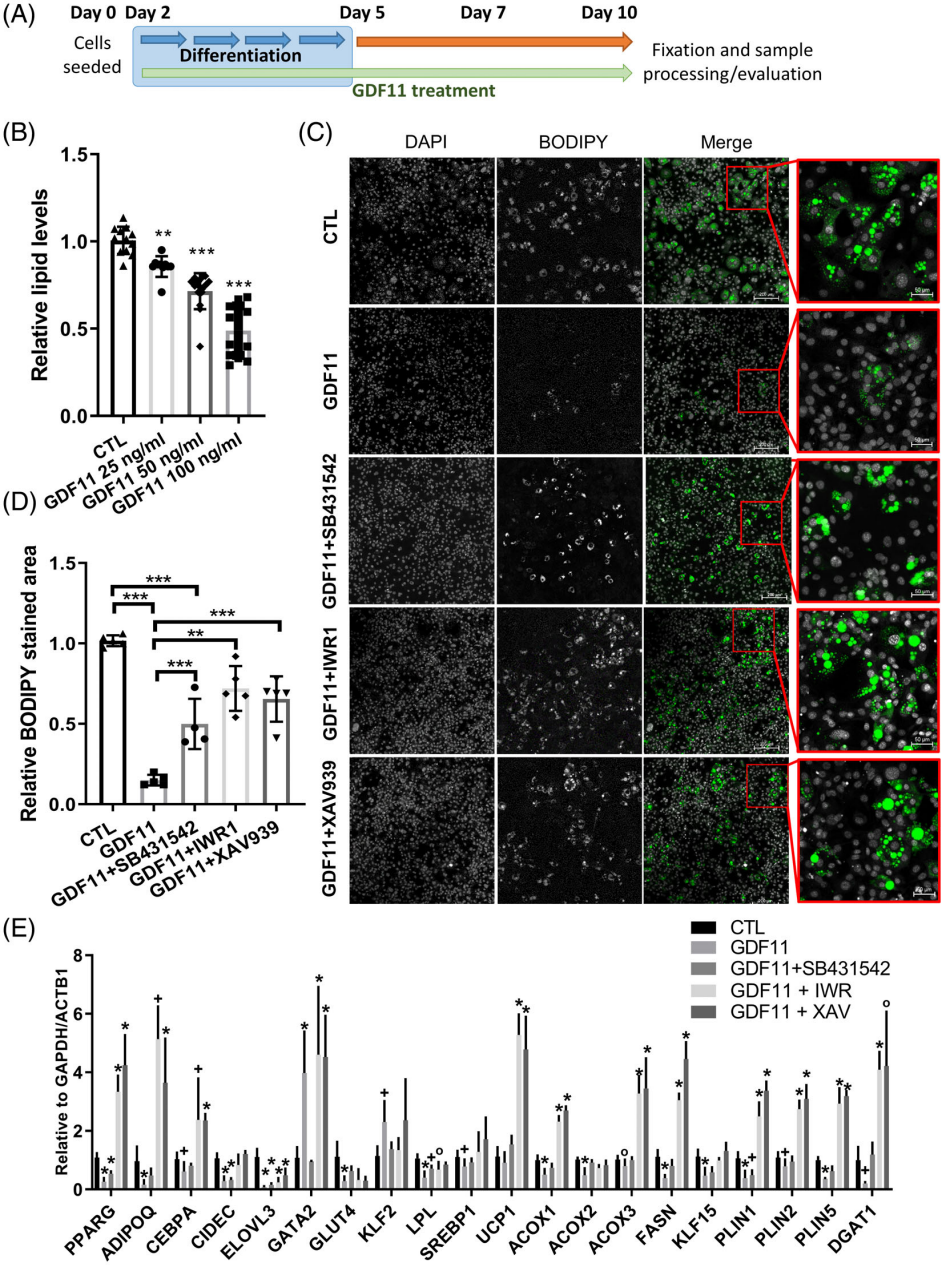
GDF11 treatment impairs 3T3‐L1 differentiation into mature adipocytes involving WNT/β‐catenin signaling. (A) Schematic figure illustrating used 3T3‐L1 cell line differentiating and treatment protocol. (B) Intracellular lipid levels quantification of BODIPY and ORO measurement in 3T3‐L1 adipocytes, that were treated with increasing doses of recombinant GDF11 (25–100 ng/ml) (at least *n* = 4 per treatment group). **p < 0.01; ***p < 0.001 (Mann–Whitney U‐test) (C) Representative image of BODIPY stained lipids in CTL, GDF11 (100 ng/ml), GDF11 (100 ng/ml) + SB431542 (50 nM), GDF11 (100 ng/ml) + IWR1 (100 nM) and GDF11 (100 ng/ml) + XAV939 (100 nM) treated 3 T3‐L1 cells during the whole differentiation protocol (10 days), respectively (Lipids‐Green, scale = 200/50 μm, respectively). (D) Intracellular lipid levels quantification of BODIPY measurement (at least *n* = 4 per treatment group). **p < 0.01; ***p < 0.001 (Mann–Whitney *U* test). (E) mRNA expression levels of selected genes involved in adipocyte differentiation and fat metabolism (at least *n* = 3 per group from different cell line passages). ^o^ *p* < 0.05; ^+^ *p* < 0.01; *p < 0.001 (Mann–Whitney *U* test, compared with CTL samples).

In order to evaluate the effect of GDF11 supplementation on adipogenic differentiation, we treated 3T3‐L1 pre‐adipocytes with graded concentrations of GDF11 (25, 50, and 100 ng/ml) during the whole differentiation period as illustrated in Figure 3A. The degree of differentiation was evaluated by BODIPY lipid staining. Increasing concentration of rGDF11 (25–100 ng/ml) proportionately and significantly diminished lipid accumulation, as assessed by quantitative photometric and microscopic analyses of BODIPY staining (Figure 3B,C). The highest dose of rGDF11 (100 ng/ml) showed the strongest inhibitory effect with up to 45% reduction (Figure 3B, C).To further validate this negative effect on adipogenic‐committed 3T3‐L1 cells, we recapitulated experiments in human SGBS pre‐adipocytes (Supplementary Figure 2A). In line with 3T3‐L1, cell viability did not reveal cytotoxic effects of GDF11 in SGBS cells (*data not shown*). Consistent with 3T3‐L1 data, differentiation of SGBS cells with rGDF11 had similar negative effect on adipogenic differentiation (Supplementary Figure 2B,C).

To confirm evidence that GDF11‐dependent SMAD2/3 activation is responsible for the decrease of adipogenesis,^42^ we inhibited ALK5 receptor with SB431542, a potent and selective ALK5 inhibitor. Addition of SB431542 (50 ng/ml) together with GDF11 (100 ng/ml) treated 3T3‐L1 and SGBS pre‐adipocytes during the whole differentiation period, significantly rescued adipogenesis and formation of mature adipocytes (Figure 3C,D and Supplementary Figure 2B,C).

Next, we evaluated mRNA expression of genes that are essential for the process of adipogenesis (PPARG, CIDEC, GATA2, KLF2, CBPA, and SREBP) and lipid storage/metabolism (PLIN1‐3, ACOX1‐3, FASN, KLF15, GLUT4, LPL, and DGAT1) as was previously described.^52^, ^54^, ^55^ Our analysis revealed significantly downregulated mRNA levels of PPARG, SREBP1, and KLF15, which are important transcription factors that promote formation of new adipocytes (Figure 3E). Moreover, GDF11 significantly increased mRNA of transcription factors GATA2 and KLF2, which are well known inhibitors of adipogenic differentiation. GDF11 treatment was also accompanied by the decreased expression of PLIN1, PLIN5, DGAT1, and FASN that are involved in lipid storage and metabolism, whose decrease mirrors lower rates of adipocyte differentiation and lipid accumulation. Co‐administration of rGDF11 and SB431542 reversed the observed changes in the mRNA expression of genes involved in adipogenesis and lipid metabolism. In particular, ALK5 inhibition decreased mRNA of GATA2 and KLF2 and at the same time significantly elevated mRNA levels of PPARG. Next, we wondered whether activation of PPARG, a master regulator of adipogenesis,^56^ could overcome GDF11 mediated‐ablation of adipogenesis. To this purpose, we used GW1929 that is a strong PPARG agonist However, its supplementation to pre‐adipocytes (100 ng/ml) together with GDF11 during the whole differentiation period did not ameliorate adipogenesis decrease, suggesting that other intracellular pathways and transcription factors are involved (Supplementary Figure 3A–C).

### 3.4 GDF11‐dependent inhibition of adipogenesis: interaction between ALK5‐SMAD2/3 signaling and WNT/β‐catenin pathways

**FIGURE 4:**
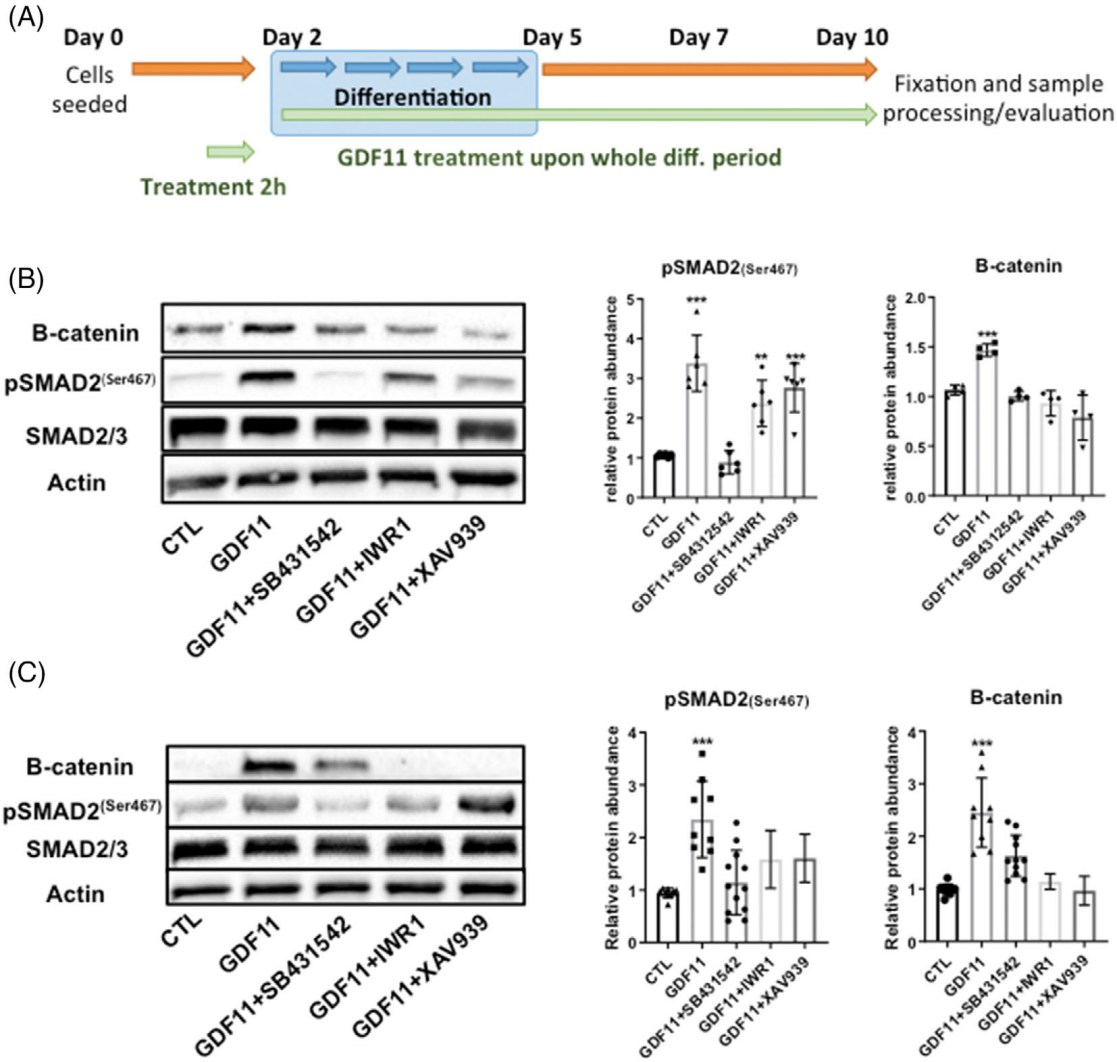
GDF11 treatment during adipogenic differentiation activates WNT/β‐catenin signaling. (A) Schematic figure illustrating 3T3‐L1 cell line treatment and differentiation protocol. Representative western blot images and quantification of pSMAD2, SMAD2/3 and B‐catenin proteins in GDF11 (100 ng/ml), SB431542 (50 nM), IWR‐1 (100 nM) and XAV939 (100 nM) treated (B) undifferentiated 3T3‐L1 cells and (C) 3T3‐L1 cells that were treated during the whole differentiation protocol (10 days, see Figure 5A) At least *n* = 6 per group from three separate cell line passages were used for the WB analysis. When using ALK5 inhibitor (SB431542, μg/ml) or B‐catenin inhibitors (IWR‐1, XAV939 both 100 nM) cells were pre‐treated for 2 h before addition of GDF11 (100 ng/ml). **p < 0.01; ***p < 0.001 (Mann–Whitney *U* test).

**FIGURE 5:**
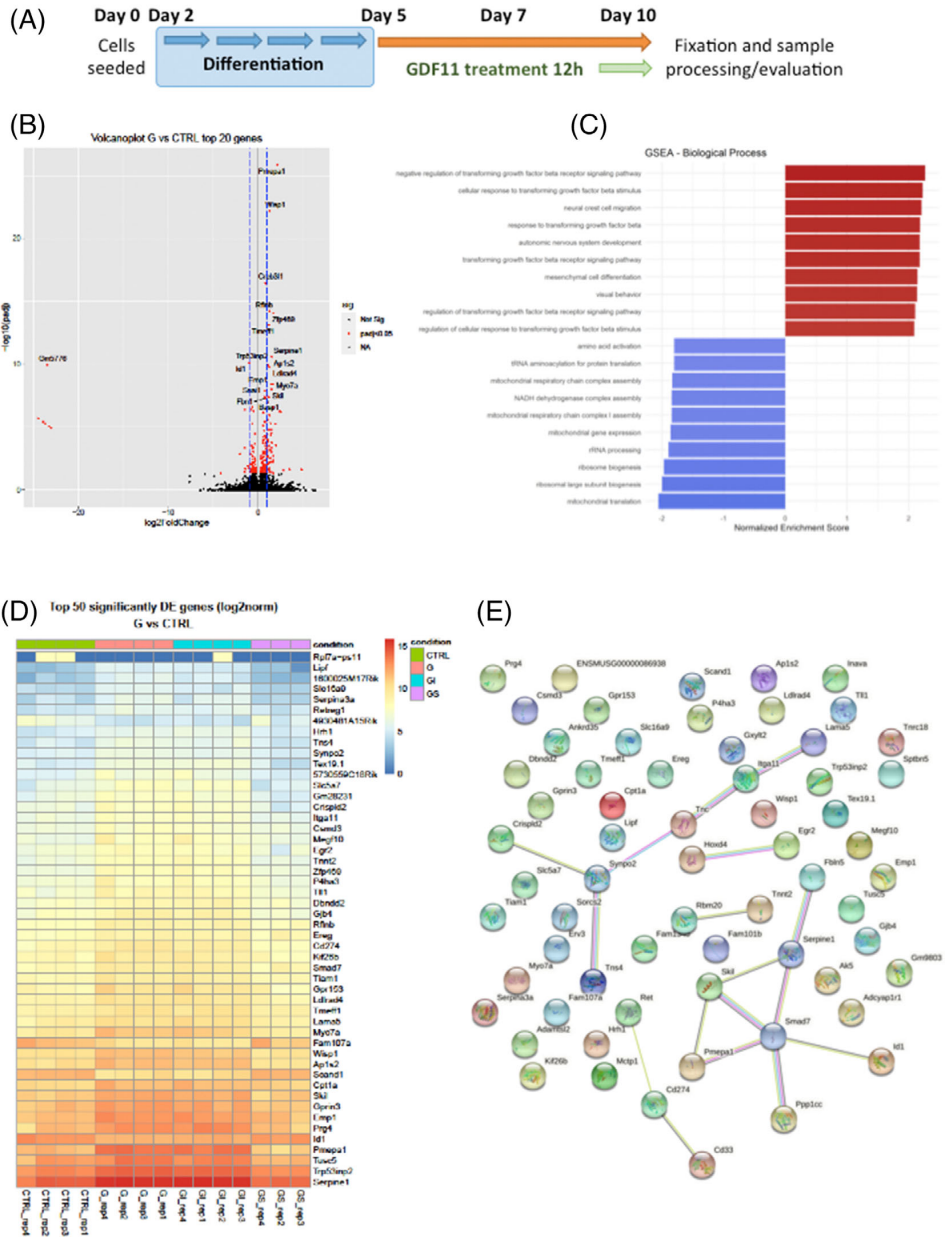
GDF11 treatment significantly affects mRNA expression levels of mature 3T3‐L1 adipocytes. (A) Schematic figure illustrating 3T3‐L1 cell line differentiating protocol and GDF11 treatment regimen. (B) Volcano plot of DESeq2 results with independent filtering. Maximum of 20 most differentially expressed genes (by adjusted *p* value of <0.05) are named. (C) Computational Gene Set Enrichment Analysis (GSEA) of our statistically significant deregulated genes to uncover the most affected cellular biological processes (D) Heat map showing differences in mRNA expression levels in mature 3T3‐l1 adipocytes treated for 12 h with GDF11 and ALK5/β‐catenin inhibitors (at least *n* = 3 per group). (E) Graphical representation of gene interactions by STRING pathway analysis of Top 50 significantly (*p* ≤ 0.01) deregulated genes. Connections between genes are indicating annotated, high confidence (>0.7) molecular interactions.

Our transcriptomic experiments in *ob/ob* mice pointed out that both TGF‐β and canonical WNT/β‐catenin signaling pathways are modulated by GDF11 in the adipose tissue. Hence, to unravel the role of these pathways and their potential crosstalk in adipogenesis, we tested inhibitors of canonical WNT/β‐catenin signaling, XAV939 and IWR1, on 3T3‐L1 pre‐adipocytes together with rGDF11 treatment. XAV939 is a potent Tankyrase (TNKS) inhibitor that antagonizes WNT via stimulation of β‐catenin degradation, whereas IWR1 (endo‐IWR1) promotes β‐catenin phosphorylation (i.e., degradation) by stabilizing Axin‐scaffolded destruction complexes.^57^Both inhibitors at used concentration of 100 nM ameliorated GDF11 mediated 3T3‐L1 adipogenic decrease, in a similar fashion compared with the ALK5 inhibition, suggesting that the observed arrest of adipocyte differentiation could be connected to WNT activation rather than to SMAD2/3 downstream signaling only (Figure 3C,D). Expression analysis of important adipogenic and lipid metabolism genes showed that after the treatment with WNT inhibitors not only a partial restoration to CTL levels (CIDEC, SREBP1, LPL, ELOVL3, and KLF15), but in most cases massive upregulation of mRNA expression levels of genes involved in adipogenesis (PPARG, CIDEC, ADIPOQ, and CBPA) and lipid metabolism (ACOX1 and 3, FASN, DGAT1 and PLIN1, 2, and 5) (Figure 3E). This upregulation can be explained by the inhibition of basal WNT during adipogenesis and adipocyte maturation, which normally suppresses/regulates differentiation of precursor cells into mature adipocytes.^58^Next, we explored links between GDF11 triggered ALK5‐SMAD2/3 signaling and WNT/β‐catenin activation using immunoblotting analysis. Firstly, we examined whether WNT/β‐catenin pathway can be activated by GDF11 treatment in undifferentiated 3T3‐L1 pre‐adipocytes. As shown in Figure 4A,B, GDF11 treatment for 2 h significantly elevated levels of intracellular β‐catenin, whereas inhibitors of ALK5 and WNT pathways decreased intracellular β‐catenin and hence restored WNT/β‐catenin signaling to basal levels. Similar results were obtained in 3T3‐L1 cells that were treated with GDF11 (100 ng/ml), ALK5 and β‐catenin inhibitors (IWR‐1 or XAV939) during the whole differentiation period (Figure 4C). In conclusion, inhibitors of ALK5 and WNT signaling rescued GDF11 mediated arrest of adipogenesis and at the same time decreased WNT signaling. Together with observation that inhibition of WNT pathway did not affected intracellular phosphorylation of SMAD2 triggered by GDF11 (Figure 4B, C), this suggests that WNT signaling—rather than SMAD2/3 downstream signaling—might be responsible for the observed adipogenic arrest.To further investigate a link between TGF‐β,WNT/β‐catenin and adipogenesis, lipid/glucose metabolism and lipid storage, we performed RNA‐Seq analysis mature 3T3‐L1 adipocytes treated with GDF11 and/or inhibitors of ALK5 (SB431542) and WNT signaling (IWR1) (Figure 5A). The analysis uncovered significant changes in the expression of 72 genes (*p* ≤ 0.05 and log2fc ≥1;−1), of which 60 were upregulated and 12 significantly downregulated. Figure 5B depicts volcano plot with marked top 20 most significantly deregulated genes. Next we performed gene set enrichment analysis (GSEA) to disclose the most affected biological processes by the treatment (Figure 5C), identifying the regulation of TGF‐β signaling, mesenchymal cell differentiation and various metabolism pathways represented by mitochondrial/respiratory genes. GDF11 treatment alone significantly affected expression patterns as evidenced by heat maps in Figure 5D, whereas ALK5 inhibition attenuated observed changes. Inhibition of WNT signaling had only minor effect on the gene expression. To examine pathway associations and link them to deregulated genes, we analyzed top 50 influenced genes in our dataset by STRING analysis tool for annotated molecular interactions of selected genes (Figure 5F).^53^ Among the top deregulated genes only few genes, Serpine1, ID1, SMAD7, Synpo2, and Skill, stood out.

### 3.5 GDF11 does not affect lipid accumulation but increases adiponectin in mature 3T3‐L1 adipocytes

**FIGURE 6:**
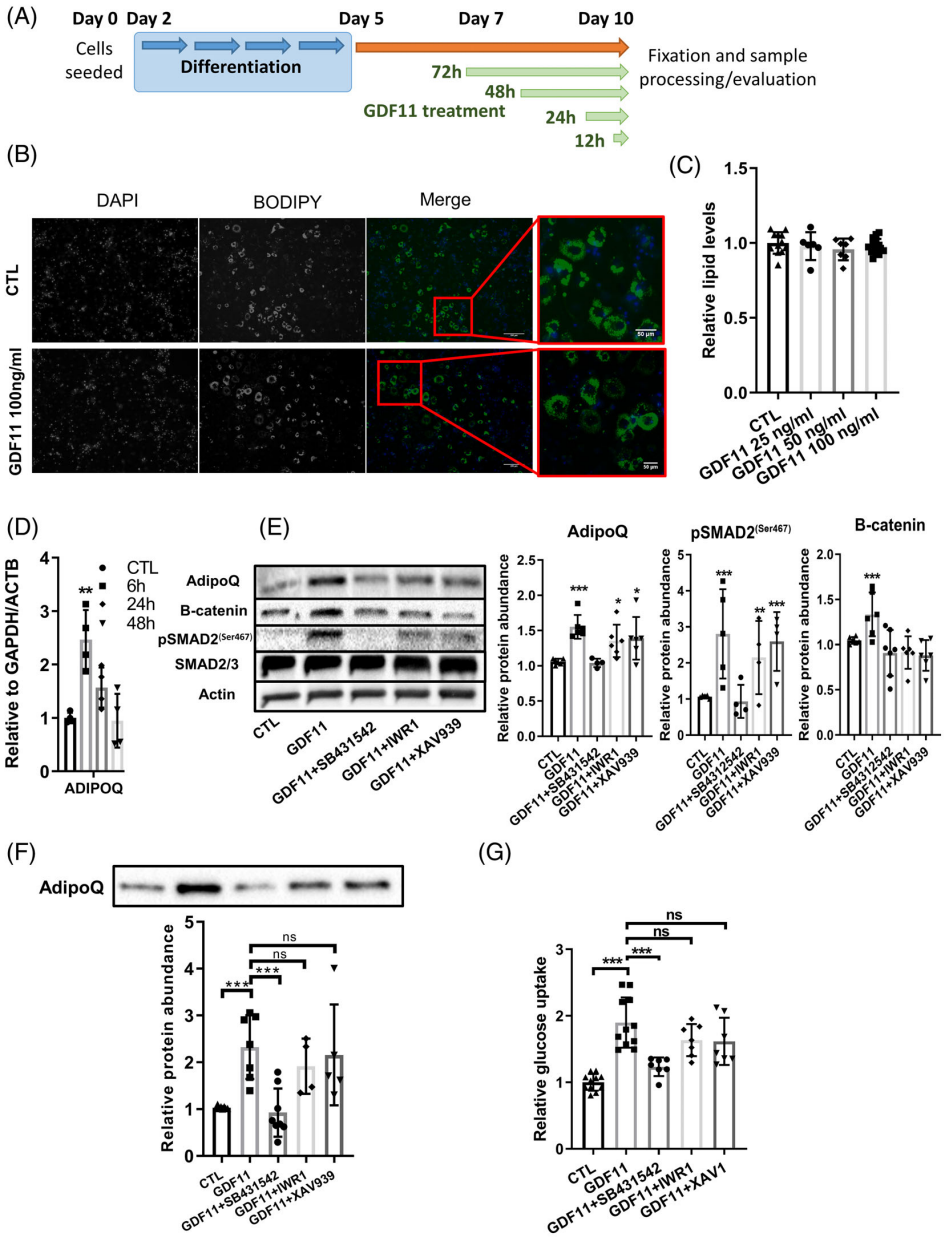
GDF11 treatment does not change adiposity in fully differentiated mature 3T3‐L1 adipocytes, but activates WNT/B‐catenin signaling and elevates adiponectin levels. (A) Schematic figure illustrating 3T3‐L1 cell line differentiating protocol and GDF11 treatment regimen. (B) Representative image of BODIPY stained lipid droplets in CTL or GDF11 (100 ng/ml) treated (for 72 h) mature 3T3‐L1 adipocytes (scale = 100 μm). (C) Quantification of intracellular lipid levels of both ORO and BODIPY measurement in mature 3T3‐L1 adipocytes (at least *n* = 5 per group). No change in adiposity was observed after treatment with successive doses of GDF11 (25, 50 and 100 ng/ml) for 72 h. (D) mRNA expression levels of adiponectin in mature 3T3‐L1 adipocytes treated with GDF11 (25 ng/ml) in time points 6, 24 and 48 h (at least *n* = 3 per treatment group from separate cell line passages; *p < 0.001). (E) Representative western blot images and quantification of adiponectin, β‐catenin and pSMAD2 protein levels in fully matured 3T3‐L1 adipocytes that were treated for the period of 24 h with GDF11 (100 ng/ml), SB431542 (50 nM), IWR‐1 (100 nM) and XAV939 (100 nM). At least *n* = 6 per group from 3 separate cell line passages were used for the WB quantification. When using ALK5 inhibitor (SB431542, 1 μg/ml) or B‐catenin inhibitors (IWR‐1, XAV939 both 100 nM) cells were pre‐treated for 2 h before addition of GDF11 (100 ng/ml). *p < 0.05; **p < 0.01; ***p < 0.001 (Mann–Whitney *U* test). (F) Representative immunoblotting image and densitometric analysis of secreted adiponectin protein into cell culture media of mature 3T3‐L1 adipocytes after GDF11 and ALK5/β‐catenin inhibitors treatment (at least *n* = 6 per treatment group from three independent experiments) ***p < 0.001 (Mann–Whitney *U* test). (G) Glucose uptake levels in mature 3T3‐L1 adipocytes treated with GDF11 for 24 h period (coupled with serum starvation). GDF11‐mediated ALK5 activation increases internalization of 2‐DG glucose from the media then CTL and GDF11 + SB431542 treated cells. Co‐treatment of mature 3T3‐L1 adipocytes with GDF11 and inhibitors of B‐catenin, IWR1 or XAV939, ameliorate glucose sensitivity, which is thus only ALK5‐SMAD2/3 dependent. ***p < 0.001 (Mann–Whitney *U* test).

As our and others in vivo experiments suggested, GDF11 can restore metabolic homeostasis and increase synthesis of hormones, notably adiponectin, from WAT.^39^, ^41^ To further corroborate these results, we treated mature 3T3‐L1 adipocytes with several doses of GDF11 (25–100 ng/ml, Figure 6A),^41^ measured lipid content, adiponectin expression and looked into signaling pathways involved.

GDF11 treatment of adipocytes for 72 h did not decrease lipids levels at any of the used dosages (25–100 ng/ml, Figure 6B,C), but significantly modulated adiponectin mRNA levels in a time dependent fashion (Figure 6D). Interestingly, GDF11 treatment elevated adiponectin mRNA with peak after 6 h of treatment and a decrease at later time points (Figure 6D). To test these results at the protein level we performed immunoblotting of mature 3T3‐L1 adipocytes and their conditioned media after the treatment with GDF11 for 24 h (Figure 6E,F). Our analysis showed increase of both intracellular and secreted adiponectin after GDF11 treatment (Figure 6E,F), whereas co‐treatment with ALK5 inhibitor decreased adiponectin to basal levels. Interestingly, adiponectin levels in the samples co‐treated with GDF11 and WNT/β‐catenin inhibitors remained elevated (Figure 6E,F), suggesting that its increased levels are exclusively connected to SMAD2/3 signaling rather than to WNT/ β‐catenin.

WAT contributes to glucose homeostasis/disposal to a smaller extent (only ~10% of whole body glucose pool) compared with skeletal muscle (up to 70% of all glucose).^59^, ^60^ As systemic GDF11 treatment can improve glucose homeostasis and insulin sensitivity in rodent obesity models, we tested internalization/uptake of 2‐deoxy glucose in mature 3T3‐L1 adipocytes after GDF11 treatment (Figure 6G). After 24 h treatment of 3T3‐L1 adipocytes with GDF11, a significant 2‐fold increase in glucose uptake was observed, whereas inhibition of ALK5‐SMAD2/3 signaling by SB431542 attenuated GDF11‐dependent increase in glucose uptake. Conversely, inhibition of WNT/ β‐catenin pathway by XAV939 or IWR1 had no significant effects on GDF11 induced elevation of glucose uptake.

## 4 DISCUSSION

GDF11 belongs to the TGF‐β superfamily and is essential for mammalian embryonic development and anterior‐posterior axis formation by regulating expression of Hox genes.^40^, ^61^, ^62^ For more than a decade, GDF11 received enormous attention for its “anti‐aging” (rejuvenating) and metabotrophic properties, which led often to opposite findings rendering this field controversial.^3^, ^24^, ^25^, ^28^, ^35^, ^36^, ^37^, ^63^, ^64^ Recent data linked GDF11 with WAT, metabolic homeostasis and adipogenic differentiation^39^, ^41^, ^42^ and yet, the underlying mechanisms remained poorly understood. Hence, we present new in vitro and in vivo mechanistic evidence of how GDF11 can inhibit adipogenic differentiation while ameliorating glucose tolerance and insulin sensitivity. SMAD2/3 pathway, downstream of TGF‐β activation, is critical in mediating inhibitory effects on adipogenesis.^22^, ^65^ For the first time, we present evidence of crosstalk and synergism between TGF‐β and canonical WNT/β‐catenin signaling pathways in the process of adipogenesis and adipocyte maturation. Treatment of obese *ob/ob* mice with GDF11 led to shrinkage of white adipocyte size, while triggering an anti‐adipogenic gene expression pattern with the involvement not only of expected TGF‐β pathway genes (CTGF, ACVR1B, NOG, etc.), but also genes specific for WNT/β‐catenin signaling and regulation (LGR5, FZD3, RSPO1, etc.). Our RNA‐seq analysis of mature 3T3‐L1 adipocytes treated with rGDF11 showed other potential novel genes that could be involved in the observed GDF11‐mediated arrest of adipogenesis and metabolic changes. In particular, overexpression of SERPINE1 and downregulation of ID1 genes, known to be associated with metabolic disorders, could represent other intracellular downstream mediators of GDF11 effects.^66^, ^67^, ^68^ Consistently, co‐treatment of pre‐adipocytes with GDF11 and WNT/β‐catenin inhibitors revealed that WNT signaling ameliorated GDF11‐mediated decrease in adipogenic differentiation and lipid accumulation of 3T3‐L1 cells, implying that the arrest of adipocyte differentiation mediated by GDF11 is due to the activation of WNT pathway rather than SMAD2/3 downstream dependent signaling. TGF‐β signaling pathway activation is linked to the crosstalk with other intracellular signaling pathways with various synergistic or antagonistic effects that in the end modulates biological outcomes.^69^, ^70^, ^71^ In fact, various TGF‐β ligands, in spite of activating the same intracellular signaling cascade dependent on SMAD2/3, can also impinge in parallel different intracellular signaling pathways resulting in variable intracellular outcomes.^72^, ^73^, ^74^ In this respect, crosstalk between TGF‐β and WNT signaling pathways has been reported, however synergy between these two pathways in the adipogenesis setup was just superficially explored in the past. For example, Lu et al. in 2013 discovered that TGF‐β treatment of mature 3T3‐L1 adipocytes resulted in the co‐activation of β‐catenin pathway and that WNT inhibition did not reverse TGF‐β1 mediated decrease of newly differentiated adipocytes.^75^ These results are discrepant with ours and can be explained by the utilization of different ligands/inhibitors used to block WNT signaling. In their study Lu et al. used soluble Fz8‐CRD protein, which acts as competitor for membrane bound frizzled receptor, which in turn inhibits WNT pathway at the top of the signaling cascade. Our findings instead suggest that activation of WNT pathway occurs downstream of the WNT receptors, most likely by other intracellular signaling machineries that stabilize β‐catenin complexes and induce appropriate gene expression.^69^, ^71^

Another important finding from our in vivo experiments is that GDF11 improves glucose homeostasis by ameliorating insulin sensitivity. These results are consistent with previous studies using *db/db* and STZ‐induced diabetic mice.^27^, ^39^, ^76^ These studies postulated two main mechanisms that might be behind observed changes. First, the overexpression of GDF11 could promote survival, differentiation and development of pancreatic β‐cell through SMAD2/3‐PI3K/AKT/FOXO1 signal pathways^27^, ^62^, ^77^ and the second possibility is that GDF11 blocks activation of macrophages and chronic tissue inflammation that plays a crucial role in the development of obesity‐related insulin resistance.^76^, ^78^, ^79^ Based on our in vitro data, we offer another explanation of how GDF11 can counteract insulin resistance and ameliorate diabetic symptoms. GDF11 treatment promotes direct stimulation of mature adipocytes that significantly increases glucose uptake by adipocytes, which can then contribute to amelioration of systemic glucose levels and improve glucose homeostasis. Even though the contribution of adipocytes to glucose whole body disposal is smaller in comparison to skeletal muscle,^59^, ^60^ many studies using knockout and transgenic mice deficient or overexpressing specific glucose transporters demonstrated the critical role of adipose tissue in glucose homeostasis.^60^, ^80^ Thus, all above‐mentioned mechanisms can synergistically coexist, because the role of GDF11 in glucose homeostasis and metabolism is likely systemic.

Next, we showed evidence that GDF11 treatment induces adiponectin expression and synthesis in mature, fully formed adipocytes directly by impinging ALK5‐SMAD2/3 signaling axis. Adiponectin is a multifunctional adipokine that regulates a number of cardiovascular and neurometabolic processes, including inflammation, glucose metabolism, fatty acid oxidation, body energy expenditure, and memory.^3^, ^81^, ^82^ Adiponectin is inversely correlated with body mass index in obese patients and calorie‐restriction diets restore its levels.^3^, ^83^, ^84^ Our research involving mature 3T3‐L1 adipocytes showed that treatment with recombinant GDF11 significantly elevated, for short period of time, mRNA levels of adiponectin, which were followed by its elevated intracellular protein synthesis upon 24 h. Moreover, elevated levels of secreted adiponectin were detected in conditioned media of mature adipocytes after GDF11 treatment. It is interesting to note that elevated levels of adiponectin were exclusively due to the activation of ALK5‐SMAD2/3 signaling axis after GDF11 treatment, whereas inhibition of WNT/β‐catenin canonical signaling did not affect levels of adiponectin, suggesting that both pathways activated by GDF11 are important in the adipogenesis process, but only ALK5‐SMAD2/3 can regulate mature white adipocytes functions. All these data are in accordance with previous findings, suggesting that elevated secretion of adiponectin can be another piece of the puzzle how GDF11 treatment regulates systemic energy and glucose/insulin homeostasis.^41^ Nonetheless, it is still unclear, to which extent the metabolic effects of GDF11 treatment contribute to increased adiponectin synthesis and increased glucose uptake by mature adipocytes.

In conclusion, GDF11 treatment improves glucose/insulin homeostasis, and decreases weight gain and white adipocyte size in obese rodents. Moreover, GDF11 mediates the inhibition of adipogenesis in pre‐adipocytes in a concentration‐dependent manner. Mechanistically, we propose that these inhibitory effects of GDF11 on adipogenesis are exerted through phosphorylation of SMAD2/3 and by activation of β‐catenin pathway. In mature 3T3‐L1 adipocytes, synthesis of adiponectin and glucose uptake were increased upon GDF11 exposure. Since the expansion of WAT mass seen in obesity involves hyperplasia, hypertrophy, inflammation, and glucose turnover in adipocytes, the beneficial effects of GDF11 therapy in obesity are likely to occur at multiple levels. Further studies are essential for the development of GDF11‐ and other metabotrophins‐based systemic therapies for obesity and related cardio‐ and neurometabolic diseases, including Alzheimer's disease.^1^, ^3^ We envisage that these therapies should overcome side effects in other organs—such as liver fibrosis,^85^, ^86^ targeting the adipose tissue in a tissue‐specific manner^87^

## AUTHOR CONTRIBUTIONS

Jan Frohlich, Kristina Kovacovicova, Manlio Vinciguerra: Conceptualization; Jan Frohlich, Kristina Kovacovicova, Marco Raffaele, Tereza Virglova, Eliska Cizkova, Jan Kucera, Francesca Bonomini, Michelino Di Rosa, Nicolas Blavet, Vaclav Hejret: Data curation; Jan Frohlich, Kristina Kovacovicova, Marco Raffaele, Michelino Di Rosa, Nicolas Blavet, Vaclav Hejret: Formal analysis; Manlio Vinciguerra: Funding acquisition; Jan Frohlich, Kristina Kovacovicova, Marco Raffaele, Tereza Virglova, Eliska Cizkova, Julie Bienertova‐Vasku, Nicolas Blavet, Vaclav Hejret: Investigation; Jan Kucera, Martin Wabitsch, George N. Chaldakov, Michelino Di Rosa, Nicolas Blavet, Vaclav Hejret: Methodology; Manlio Vinciguerra: Project administration; Jan Kucera, Martin Wabitsch, Julie Bienertova‐Vasku: Resource; Michelino Di Rosa: Software; Julie Bienertova‐Vasku, Rita Rezzani, George N. Chaldakov, Anton B. Tonchev, Manlio Vinciguerra: Supervision; Marion Peyrou: Validation; Michelino Di Rosa, Nicolas Blavet, Vaclav Hejret Visualization; Jan Frohlich, Marco Raffaele, Marion Peyrou, Manlio Vinciguerra Roles/Writing—original draft; Jan Frohlich, Marco Raffaele, Rita Rezzani, George N. Chaldakov, Anton B. Tonchev, Manlio Vinciguerra: Writing—review & editing.

## FUNDING INFORMATION

This study was supported by the European Social Fund and European Regional Development Fund—Project MAGNET (No. CZ.02.1.01/0.0/0.0/15_003/0000492, to Manlio Vinciguerra) and by The European Commission Horizon 2020 Framework Program (Project 856871—TRANSTEM, to Manlio Vinciguerra).

## CONFLICT OF INTEREST

The authors declare no conflict of interest.

## Supporting information


**SUPPLEMENTARY TABLE 1** Human and murine primer sequences used in this study

**SUPPLEMENTARY FIGURE 1** In vivo experimental setup using *ob/ob* mice. Two experimental groups were injected daily (14 days) with either GDF11 (0.1 mg/kg) or saline (*n* = 12 per group).
**SUPPLEMENTARY FIGURE 2**. GDF11 treatment compromises SGBS cells differentiation into adipocytes. (A) Schematic representation illustrating SGBS cell line differentiating and treatment protocol used in this study. (B) Representative image of BODIPY stained lipid droplets in CTL, GDF11 (100 ng/ml) and GDF11 (100 ng/ml) + SB431542 (50 nM) treated SGBS cells during the whole differentiation period (18 days) (Lipids‐green, Nuclei = white, scale = 100/50 μm). (C) Intracellular lipid levels quantification by BODIPY measurement of SGBS cells as in B (*n* = 4 per treatment group). *p < 0.05; **p < 0.01 (Mann–Whitney *U* test).
**SUPPLEMENTARY FIGURE 3**. Co‐treatment of 3T3‐L1 cells with GDF11 and potent PPARγ agonist GW1929 does not rescue GDF11 mediated decrease of adipogenic differentiation. (A) Schematic figure illustrating 3T3‐L1 cell line differentiation and treatment protocol used in this experiment. (B) Representative image of BODIPY stained lipid droplets in CTL, GDF11 (100 ng/ml) and GDF11 (100 ng/ml) + GW1929 (100 nM) treated 3T3‐L1 cells during the whole differentiation period (10 days) (Lipids‐green, Nuclei = white, scale = 100/50 μm). Decrease of PPARγ expression is not the main mechanism of GDF11 action against adipogenic differentiation, this process is more complex and other pathways, mechanisms and transcription factors may be involved. (C) Intracellular lipid levels quantification by BODIPY measurement as in B (at least *n* = 5 per group). ***p < 0.001 (Mann–Whitney *U* test).

**APPENDIX S1** Supporting information

**APPENDIX S2** Supporting information

## Data Availability

All data are available upon request to the corresponding author.
