# Supplementary material for: GDF11 inhibits adipogenesis and improves mature adipocytes metabolic function via WNT/β‐catenin and ALK5/SMAD2/3 pathways
Source: Cell Prolif. 2022 Aug 3;55(10):e13310. doi: 10.1111/cpr.13310 (PMC9528760; doi:10.1111/cpr.13310)
Supplement: Supplementary file 2 — SUPPLEMENTARY FIGURE 1 In vivo experimental setup using ob/ob mice. Two experimental groups were injected daily (14 days) with either GDF11 (0.1 mg/kg) or saline (n = 12 per group). SUPPLEMENTARY FIGURE 2. GDF11 treatment compromises SGBS cells differentiation into adipocytes. (A) Schematic representation illustrating SGBS cell line differentiating and treatment protocol used in this study. (B) Representative image of BODIPY stained lipid droplets in CTL, GDF11 (100 ng/ml) and GDF11 (100 ng/ml) + SB431542 (50 nM) treated SGBS cells during the whole differentiation period (18 days) (Lipids‐green, Nuclei = white, scale = 100/50 μm). (C) Intracellular lipid levels quantification by BODIPY measurement of SGBS cells as in B (n = 4 per treatment group). *p < 0.05; **p < 0.01 (Mann–Whitney U test). SUPPLEMENTARY FIGURE 3. Co‐treatment of 3T3‐L1 cells with GDF11 and potent PPARγ agonist GW1929 does not rescue GDF11 mediated decrease of adipogenic differentiation. (A) Schematic figure illustrating 3T3‐L1 cell line differentiation and treatment protocol used in this experiment. (B) Representative image of BODIPY stained lipid droplets in CTL, GDF11 (100 ng/ml) and GDF11 (100 ng/ml) + GW1929 (100 nM) treated 3T3‐L1 cells during the whole differentiation period (10 days) (Lipids‐green, Nuclei = white, scale = 100/50 μm). Decrease of PPARγ expression is not the main mechanism of GDF11 action against adipogenic differentiation, this process is more complex and other pathways, mechanisms and transcription factors may be involved. (C) Intracellular lipid levels quantification by BODIPY measurement as in B (at least n = 5 per group). ***p < 0.001 (Mann–Whitney U test). [file CPR-55-e13310-s002.pptx]

## Slide 1
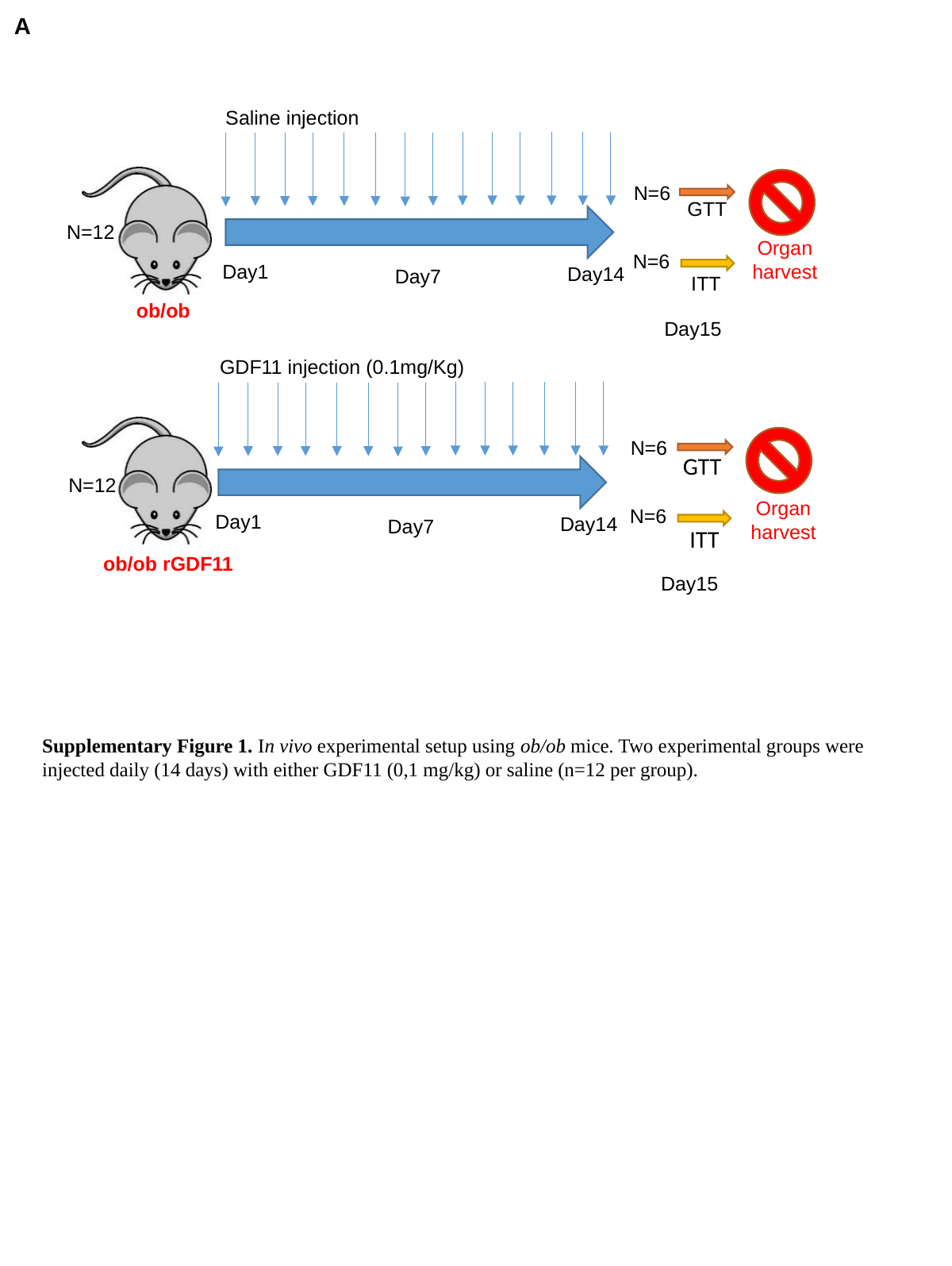

A
Saline injection
Day1
Day14
Day7
 ob/ob
N=6
GTT
N=12
Organ harvest
N=6
ITT
Day15
GDF11 injection (0.1mg/Kg)
Day1
Day14
Day7
N=6
GTT
N=12
Organ harvest
N=6
ITT
Day15
 ob/ob rGDF11
Supplementary Figure 1. In vivo experimental setup using ob/ob mice. Two experimental groups were injected daily (14 days) with either GDF11 (0,1 mg/kg) or saline (n=12 per group).

## Slide 2
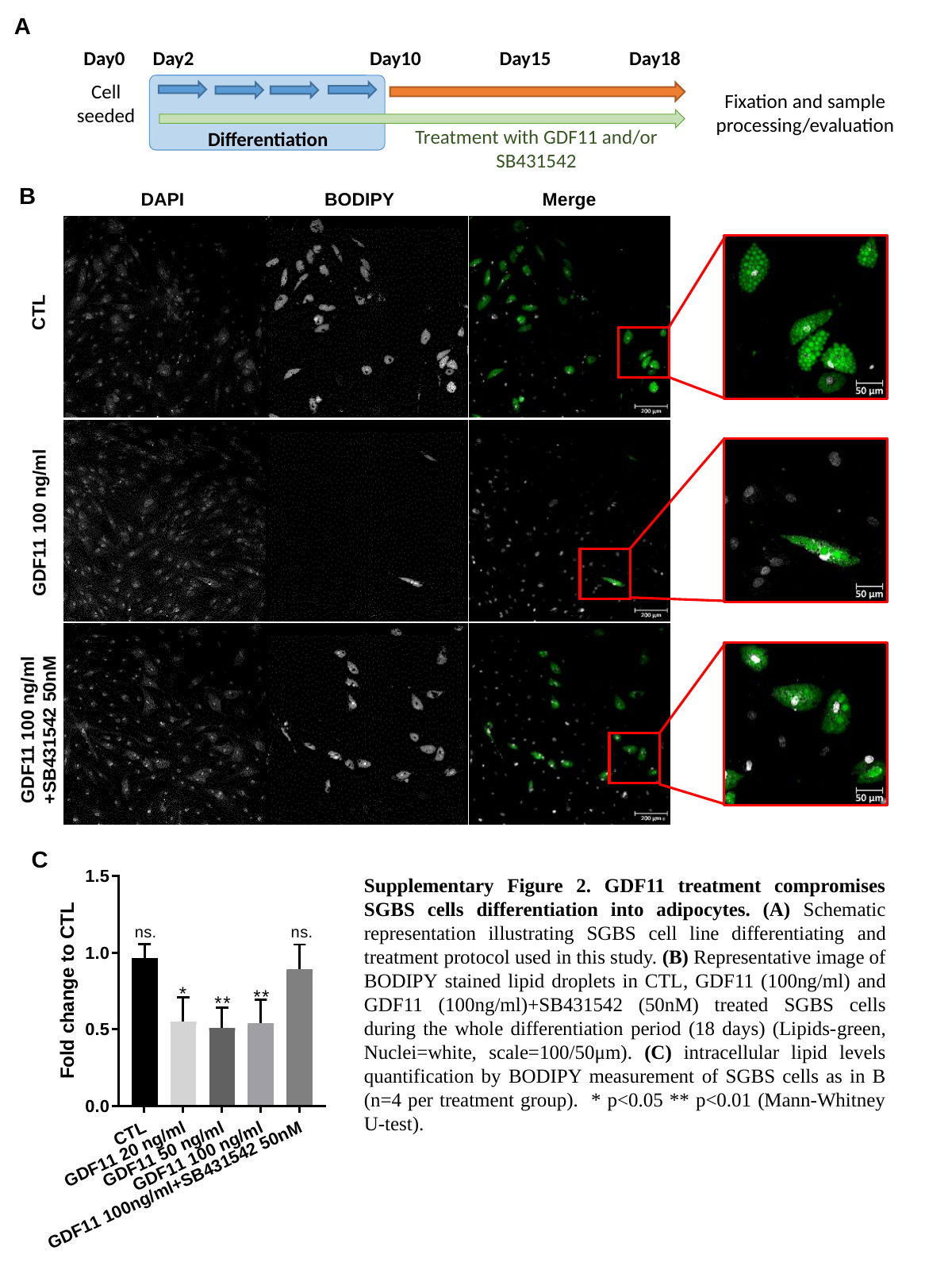

A
Day0
Day2
Day10
Day15
Day18
Cell seeded
Fixation and sample processing/evaluation
Treatment with GDF11 and/or SB431542
Differentiation
B
DAPI
BODIPY
Merge
CTL
GDF11 100 ng/ml
GDF11 100 ng/ml
+SB431542 50nM
C
Supplementary Figure 2. GDF11 treatment compromises SGBS cells differentiation into adipocytes. (A) Schematic representation illustrating SGBS cell line differentiating and treatment protocol used in this study. (B) Representative image of BODIPY stained lipid droplets in CTL, GDF11 (100ng/ml) and GDF11 (100ng/ml)+SB431542 (50nM) treated SGBS cells during the whole differentiation period (18 days) (Lipids-green, Nuclei=white, scale=100/50μm). (C) intracellular lipid levels quantification by BODIPY measurement of SGBS cells as in B (n=4 per treatment group). * p<0.05 ** p<0.01 (Mann-Whitney U-test).

## Slide 3
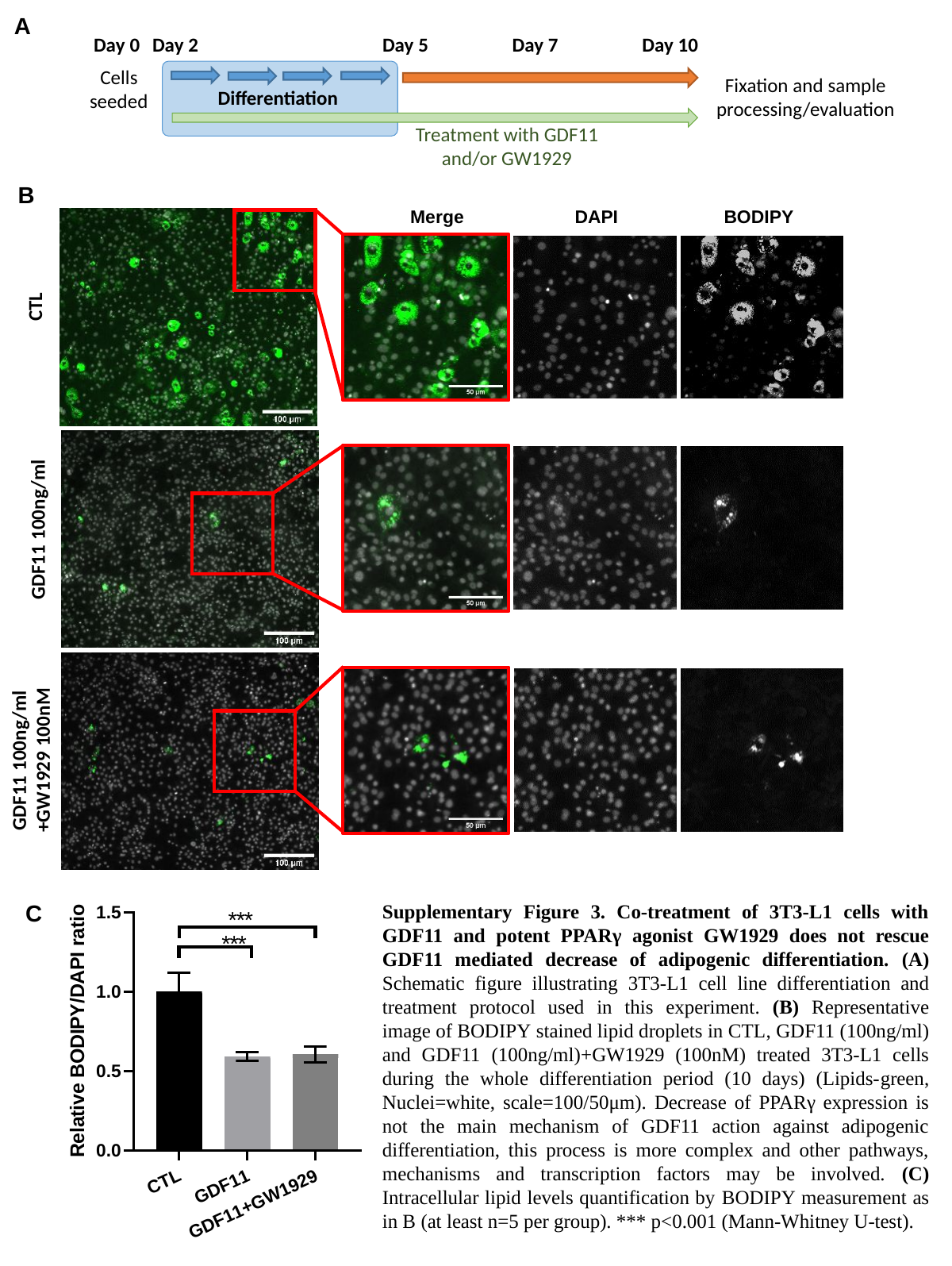

A
Day 0
Day 2
Day 5
Day 7
Day 10
Cells seeded
Fixation and sample processing/evaluation
Differentiation
Treatment with GDF11 and/or GW1929
B
Merge
DAPI
BODIPY
CTL
GDF11 100ng/ml
GDF11 100ng/ml +GW1929 100nM
C
Supplementary Figure 3. Co-treatment of 3T3-L1 cells with GDF11 and potent PPARγ agonist GW1929 does not rescue GDF11 mediated decrease of adipogenic differentiation. (A) Schematic figure illustrating 3T3-L1 cell line differentiation and treatment protocol used in this experiment. (B) Representative image of BODIPY stained lipid droplets in CTL, GDF11 (100ng/ml) and GDF11 (100ng/ml)+GW1929 (100nM) treated 3T3-L1 cells during the whole differentiation period (10 days) (Lipids-green, Nuclei=white, scale=100/50μm). Decrease of PPARγ expression is not the main mechanism of GDF11 action against adipogenic differentiation, this process is more complex and other pathways, mechanisms and transcription factors may be involved. (C) Intracellular lipid levels quantification by BODIPY measurement as in B (at least n=5 per group). *** p<0.001 (Mann-Whitney U-test).
