# Supplementary material for: GDF11 inhibits adipogenesis and improves mature adipocytes metabolic function via WNT/β‐catenin and ALK5/SMAD2/3 pathways
Source: Cell Prolif. 2022 Aug 3;55(10):e13310. doi: 10.1111/cpr.13310 (PMC9528760; doi:10.1111/cpr.13310)
Supplement: Supplementary file 4 — APPENDIX S2 Supporting information [file CPR-55-e13310-s003.docx]

*RNA-Sequencing (RNA-Seq)*

Indexed libraries were prepared from 250 ng purified RNA using a NEB Next Ultra II Directional RNA Library Prep Kit with polyA selection (Illumina, UK), according to the manufacturer's instructions. The libraries were pooled in equimolar amounts, subjected to cluster generation and sequenced on an Illumina MiSeq System (Illumina) in a 1x75 format or Illumina NovaSeq sequencer (run length 1x161 nt). Quality check of raw paired-end fastq reads was carried out by FastQC ^1^.The adapters and quality trimming of raw fastq reads was performed using Trimmomatic v0.36 ^2^ with settings CROP:250 LEADING:3 TRAILING:3 SLIDINGWINDOW:4:5 MINLEN:35 and an adapter file containing (AGATCGGAAGA). Trimmed RNA-Seq reads were mapped against the mouse genome (mm38) and Ensembl GRCm38 v.93 annotation using STAR v2.7.3a ^3^ as splice-aware short read aligner and default parameters except --outFilterMismatchNoverLmax 0.1 and --twopassMode Basic. Quality control after alignment concerning the number and percentage of uniquely- and multi-mapped reads, rRNA contamination, mapped regions, read coverage distribution, strand specificity, gene biotypes and PCR duplication was performed using several tools namely RSeQC v2.6.2 ^4^, Picard toolkit v2.18.27^5^, Qualimap v.2.2.2 ^6^ and BioBloom tools v 2.3.4-6-g433f ^7^. The differential gene expression analysis was calculated based on the gene counts produced using RSEM tool v1.3.1 ^8^ and further analyzed by Bioconductor package DESeq2 v1.20.0 ^9^. Data generated by DESeq2 with independent filtering were selected for the differential gene expression analysis due to its conservative features and to avoid potential false positive results. Genes were considered as differentially expressed based on a cut-off of adjusted p-value ≤ 0.05 and log2(fold-change) ≥1 or ≤-1. Clustered heatmaps were generated from selected top differentially regulated genes using R package pheatmap v1.0.10 ^10^, volcano plots were produced using ggplot v3.3.3 package ^11^ and MA plots were generated using ggpubr v0.4.0 package ^12^. Functional and pathway enrichment analyses on differentially expressed genes were made using the Ingenuity Pathway Analysis (IPA, spring 2018 release, QIAGEN Inc., https://www.qiagenbioinformatics.com/products/ingenuity-pathway-analysis) software package. Genes were considered differentially expressed between groups if their expression values significantly differed by >2 fold with a P ≤0.05. Correlations between GDF11 gene expression levels and other genes were determined by Pearson χ2 test for categorical variables (variables with limited or fixed, number of possible values).

*Histological and immunofluorescence analyses*

The slides were then processed by hematoxylin & eosin (H&E) staining for histological evaluation as described previously ^13-15^. The infiltration of the liver with fat/steatosis was assessed using the ImageJ software analysis program on H&E-stained sections by counting 200 randomly chosen lipid droplets per sample/mouse (n=6 mice per group, GDF11/CTL groups) and selecting their diameter (um) and area (um^2^) at 400x magnification. The morphometrical analyses were performed by two different observers blinded to the study conditions, using Image Pro Premier 9.1 (MediaCybernetics Inc., OR, USA). After washing three times with PBS, slides were counterstained with DAPI (1 µg/ml) solution and mounted in Mowiol hardening media. Images were captured using an Axio scan Z.1 equipped with a Hamamatsu ORCA-Flash 4.0 camera and ImageJ software analysis program was used to evaluate all immunofluorescence images. Macrophage infiltration or αSMA/ACTA2abundance in mouse liver samples was evaluated as % of positive area per frame at least in fifteen blindly chosen fields-of-view from each sample at 200x magnification. To evaluate the degree of apoptosis rate, Click-iT TUNEL AF647 Imaging Assay (C10247, Thermofisher) was used according to manufacturer’s instruction. Slides were then scanned using Axio scan Z.1 and image analysis was done by ImageJ software, where, at least six blindly chosen fields-of-view were evaluated.

*References*

1. Andrews S. FastQC. In:2010.

2. Bolger AM, Lohse M, Usadel B. Trimmomatic: a flexible trimmer for Illumina sequence data. *Bioinformatics.* 2014;30(15):2114-2120.

3. Dobin A, Davis CA, Schlesinger F, et al. STAR: ultrafast universal RNA-seq aligner. *Bioinformatics.* 2013;29(1):15-21.

4. Wang L, Wang S, Li W. RSeQC: quality control of RNA-seq experiments. *Bioinformatics.* 2012;28(16):2184-2185.

5. Broad-Institute. “Picard Toolkit” Broad Institute, GitHub Repository. <http://broadinstitute.github.io/picard/>. 2018.

6. Okonechnikov K, Conesa A, García-Alcalde F. Qualimap 2: advanced multi-sample quality control for high-throughput sequencing data. *Bioinformatics.* 2016;32(2):292-294.

7. Chu J, Sadeghi S, Raymond A, et al. BioBloom tools: fast, accurate and memory-efficient host species sequence screening using bloom filters. *Bioinformatics.* 2014;30(23):3402-3404.

8. Li B, Dewey CN. RSEM: accurate transcript quantification from RNA-Seq data with or without a reference genome. *BMC Bioinformatics.* 2011;12(1):323.

9. Love MI, Huber W, Anders S. Moderated estimation of fold change and dispersion for RNA-seq data with DESeq2. *Genome Biology.* 2014;15(12):550.

10. Kolde R. "Package ‘pheatmap’." R Package 1.7. 2015.

11. Wickham H. ggplot2. 2011;3(2):180-185.

12. Kassambara A. "ggpubr:“ggplot2” based publication ready plots." R package version 0.1 7. 2018.

13. Benegiamo G, Mazzoccoli G, Cappello F, et al. Mutual Antagonism between Circadian Protein Period 2 and Hepatitis C Virus Replication in Hepatocytes. *PLoS ONE.* 2013;8(4).

14. Borghesan M, Fusilli C, Rappa F, et al. DNA Hypomethylation and Histone Variant macroH2A1 Synergistically Attenuate Chemotherapy-Induced Senescence to Promote Hepatocellular Carcinoma Progression. *Cancer research.* 2016;76(3):594-606.

15. Vinciguerra M, Veyrat–Durebex C, Moukil MA, Rubbia–Brandt L, Rohner–Jeanrenaud F, Foti M. PTEN Down-Regulation by Unsaturated Fatty Acids Triggers Hepatic Steatosis via an NF-κBp65/mTOR-Dependent Mechanism. *Gastroenterology.* 2008;134(1):268-280.
